# Separating Out Pulmonary Sequestration

**DOI:** 10.7759/cureus.53190

**Published:** 2024-01-29

**Authors:** Linda H Pham, Yassine Hamdaoui, Ghazarian Zeron, Ahmed El-Bershawi, Ahmad Alazzeh

**Affiliations:** 1 Internal Medicine, HCA Riverside, Riverside, USA; 2 Internal Medicine, University Sidi Mohamed Ben Abdallah Faculty of Medicine and Pharmacy of Fes, Fes, MAR; 3 Pulmonary Critical Care, HCA Riverside, Riverside, USA

**Keywords:** congenital lung abnormalities, pulmonary disease, pulmonary sequestration, recurrent pneumonia, intralobar pulmonary sequestration

## Abstract

Pulmonary sequestration (PS) is a rare congenital anomaly that accounts for 1% to 6% of all pulmonary malformations at birth. It is characterized by a focal area of pulmonary tissue that does not have direct communication with the tracheobronchial tree and does not get blood supply from the pulmonary circulation. We present the case of a 28-year-old female with a history of recurrent pulmonary infections who was found to have intralobar sequestration and underwent curative surgical excision. Because pulmonary sequestration is commonly misdiagnosed, as it can mimic other conditions on chest X-rays, this case illustrates the importance of recognizing pulmonary sequestration as a separate entity and diagnosing/treating it appropriately.

The patient presented to the hospital with a one-week history of upper chest pain. Chest radiograph showed mild hyperinflated right lung. Computed tomography angiogram (CTA) revealed an 8.9 x 8.3 cm area of hyper-lucency and decreased normal lung architecture in the right lower lobe with an aberrant arterial blood supply suggestive of intralobar pulmonary sequestration. The patient was referred to cardiothoracic surgery and underwent preoperative outpatient pulmonary function testing, which was unremarkable. The patient subsequently underwent successful robotic resection of the right lower lobe sequestration and the pathology report confirmed PS.

A diagnosis of pulmonary sequestration is commonly missed, as it can mimic other conditions on chest X-rays. It can present as a solitary nodule or mass, cystic lesion, consolidation, or an air-fluid level. The period between symptom onset and diagnosis is typically more than five years. While digital subtraction angiography is considered the gold standard for imaging, CTA is now preferred because it allows for clear visualization of lung parenchyma and vascular assisting in surgical planning. For our patient, CTA demonstrated a prominent tubular vessel, which showed less enhancement than the opacified pulmonary artery and pulmonary veins, suggestive of an abnormal vascular supply for the right lower lobe sequestration. Management of intralobar sequestration is curative surgical excision. Both video-assisted thoracoscopic surgery (VATS) and posterolateral thoracotomy are viable options for resection. It should be noted that in symptomatic patients, it is recommended to proceed with surgical resection. However, in asymptomatic individuals with intralobar sequestration (ILS), surgical resection is not required but could be considered as prophylaxis to prevent recurrent infections. Asymptomatic individuals with extralobar sequestration (ELS), on the other hand, should undergo serial monitoring as non-operative management is appropriate.

This case highlights the importance of including pulmonary sequestration, especially intralobar sequestration in the differential diagnosis of recurrent localized pulmonary infections, especially in a patient who is otherwise healthy. Although rare, it is important to consider this congenital anomaly when evaluating patients with recurrent localized pulmonary infections, chest pain, or hemoptysis.

## Introduction

Pulmonary sequestration (PS) is a rare congenital anomaly that accounts for 1% to 6% of pulmonary malformations at birth. It is characterized by a focal area of pulmonary tissue that does not have direct communication with the tracheobronchial tree and does not get blood supply from the pulmonary circulation [1]. PS is classically classified into two types: intralobar sequestration (ILS) and extralobar sequestration (ELS). In ILS, the sequestration shares the visceral pleura with the functioning lung. On the other hand, ELS has a separate visceral pleura maintaining complete separation from the adjacent lung [2]. 

Intralobar sequestration is frequently asymptomatic and often diagnosed incidentally in adults during chest CT examinations. Nearly 50% of adults with intralobar sequestration exhibit no symptoms. When symptoms are present, the most prevalent presentation is recurrent pulmonary infections, such as pneumonia, affecting one particular lung segment. Additional symptoms may include persistent exertional shortness of breath, hemoptysis, chronic cough, or even back pain [2].

We present the case of a 28-year-old female with a history of recurrent pulmonary infections who was found to have intralobar sequestration on imaging. This case is valuable as pulmonary sequestration is a rare diagnosis that can easily be missed and requires surgical resection as curative treatment. If pulmonary sequestration is not appropriately diagnosed, patients could continue getting recurrent infections and subsequent antibiotic courses without treating the true source of the issue. All imaging and case details presented below were obtained with the patient's written consent.

## Case presentation

**Figure 1:**
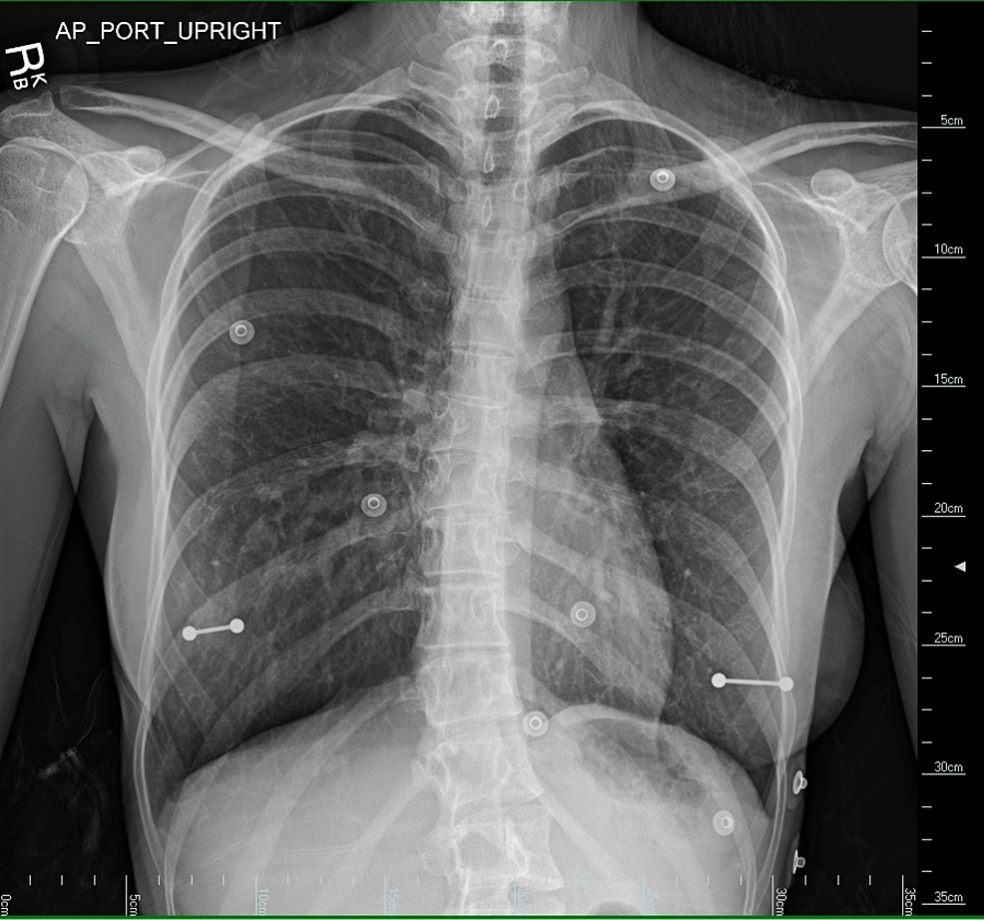
Chest radiograph of a 28-year-old woman showing subtle right lung hyperinflation

The patient was a 28-year-old female with a history of right hip osteoarthritis, thoracogenic scoliosis of the thoracolumbar region, and recurrent pulmonary infections since childhood. She presented to the hospital with a one-week history of upper chest pain. A chest radiograph was done, but it was unremarkable apart, from a mild hyperinflated right lung (Figure 1).

**Figure 2:**
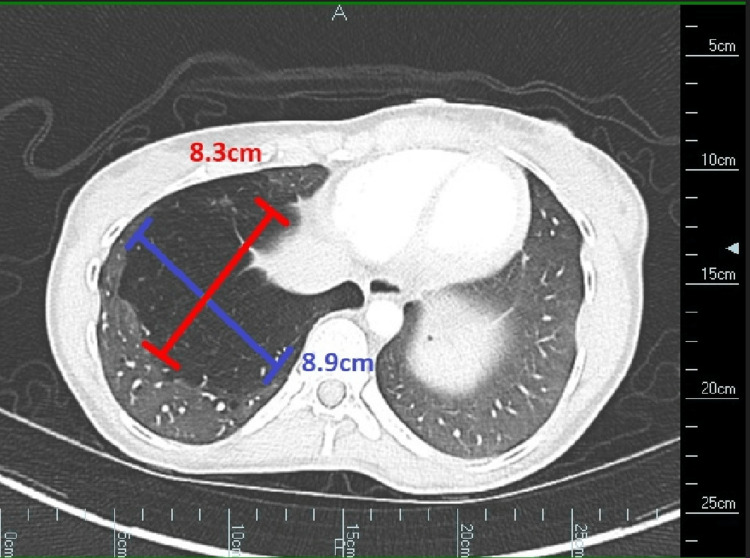
CTA of a 28-year-old female showing an 8.9 x 8.3 cm area of hyperlucency and decreased normal lung architecture in the right lower lobe CTA: computed tomography angiogram

Computed tomography angiogram (CTA) was ordered to evaluate for pulmonary embolism, which was negative. However, the CTA axial view revealed an 8.9 x 8.3 cm area of hyperlucency and decreased normal lung architecture in the right lower lobe, consistent with intralobar sequestration (Figure 2).

**Figure 3:**
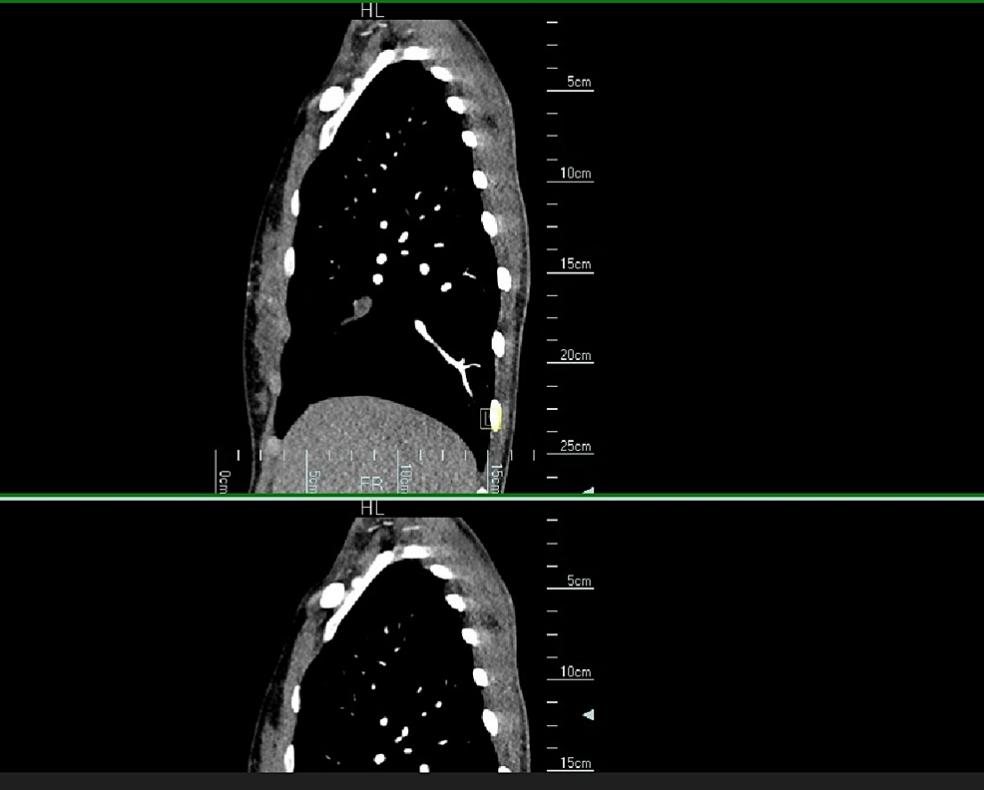
CTA sagittal view of a 28-year-old female showing a prominent tubular vessel that shows less enhancement than the opacified pulmonary artery and pulmonary veins consistent with an aberrant arterial supply of pulmonary sequestration CTA: computed tomography angiogram

CTA sagittal view (Figure 3) further revealed an aberrant arterial blood supply of the intralobar pulmonary sequestration.

The patient reported a history of recurrent pulmonary infections, including pneumonia and bronchitis, since childhood. She denied tobacco use but reported smoking marijuana weekly from ages 19-28. She had no personal or family history of congenital disorders, asthma, allergies, or anaphylaxis. She only used albuterol as needed, which helped her symptoms of cough and shortness of breath. Physical examination was unremarkable, and vital signs were within normal limits.

The patient was referred to cardiothoracic surgery and was scheduled for preoperative pulmonary function testing (PFT). Antibiotics were not prescribed at the time due to no active signs of infection. PFTs were performed and were unremarkable.

The patient underwent successful robotic resection of the right lower lobe sequestration, and her postoperative recovery was uneventful with no complications. The pathology report confirmed the presence of sequestration in the right lung lower lobe. Additionally, it indicated chronic bronchitis/bronchiolitis, mucostasis, and cystic parenchymal maldevelopment. The report also noted the presence of necrotizing granuloma, but special stains for microorganisms yielded negative results. Furthermore, pleural adhesions were observed.

## Discussion

Pulmonary sequestration is defined as a focal area of pulmonary tissue that does not have direct communication with the tracheobronchial tree and does not get blood supply from the pulmonary circulation. Pulmonary sequestration is classified as either intralobar sequestration or extralobar sequestration with ILS accounting for about 75% of cases. An intralobar sequestration is sharply demarcated from the adjacent normal lung parenchyma, with no pleura while ELS has its own visceral pleura. ILS typically involves the replacement of lung parenchyma by chronic inflammation with mucus accumulation, whereas ELS causes the bronchi, bronchioles, and alveoli to be enlarged and irregular. ELS typically is diagnosed during the neonatal period with patients presenting with recurrent infections, respiratory distress, and cyanosis, whereas approximately 60% of intralobar sequestrations are diagnosed in individuals under age 20. Furthermore, ILS is rarely ever found in individuals older than 50 years [2]. ILS is often asymptomatic or it presents as recurrent pneumonia. Missed diagnosis is very common in pulmonary sequestration patients as the period between symptom onset and diagnosis is typically more than five years [3]. Our patient endorsed an extensive history of recurrent respiratory infections, including pneumonia, since childhood. However, she was never given any explanation for why she continued to have these periodic respiratory infections. This is likely because she had never had any chest imaging aside from chest X-rays before this encounter. Pulmonary sequestration can mimic other conditions and is suggested by chest radiography in patients with recurrent pneumonia or localized bronchiectasis. Pulmonary sequestration can be misdiagnosed as a solitary nodule or mass. It can also present as a cystic lesion, consolidation, or occasionally an air-fluid level, which can be misdiagnosed as pneumonia, pyogenic abscess, infected bullae, fungal infection, or even necrotic tumor [4]. Our patient’s chest X-ray was unremarkable and did not display any of the aforementioned presentations.

Digital subtraction angiography has traditionally been the gold standard for imaging, but multi-planar CT with three-dimensional reconstruction and MR angiography are now considered better diagnostic tools. These modalities are both less invasive and more cost-efficient than digital subtraction angiography. As a non-invasive imaging technique, multidetector computed tomography angiography has been found to be a useful tool in aiding diagnostic decision-making for pulmonary sequestration. It allows for clear visualization of the related parenchymal characteristics, arterial supply, and venous drainage features, which can assist in the planning of surgical strategies with a high level of confidence [5]. As the authors in Sadasivan Nair et al. concluded, imaging is of great importance to properly identify the anomalous arterial supply to the sequestration so as to avoid the anomalous systemic arterial feeding vessel during the surgical resection [6]. CT chest with contrast can reveal various presentations of the sequestered lung tissue, including bronchiectasis, a cyst, localized atelectasis, a lamellar lesion, or a capsulated lesion with an air-fluid level. In addition, emphysematous blebs in abnormal locations with non-functioning lung tissue have also been reported as a presentation of pulmonary sequestration [7].

The imaging goals are twofold: to exclude other thoracic pathologies and to demonstrate the presence of an abnormal arterial supply. Definitive diagnosis of pulmonary sequestration requires confirmation of the abnormal vascular supply and venous drainage to the sequestered lung tissue [2]. For our patient, CT angiography demonstrated a prominent tubular vessel that showed less enhancement than the opacified pulmonary artery and pulmonary veins, suggestive of an abnormal vascular supply for the right lower lobe sequestration.

Mild to moderate obstructive disease has been identified in 8.8% of patients with pulmonary sequestration [3]. However, in our case, the patient did not have obstructive disease.

The management of intralobar sequestration is surgical excision, which is curative. Surgical resection was found to be well-tolerated from a pulmonary function perspective [8]. Both video-assisted thoracoscopic surgery (VATS) and posterolateral thoracotomy are viable options for pulmonary sequestration resection. VATS may offer benefits over thoracotomy, including a shorter postoperative hospital stay [3]. It should be noted that in symptomatic patients, it is recommended to proceed with surgical resection. However, in asymptomatic individuals with ILS, surgical resection is not required but could be considered as prophylaxis to prevent recurrent infections [3]. Asymptomatic individuals with ELS, on the other hand, should undergo serial monitoring as nonoperative management is appropriate [9]. Symptomatic patients with ELS should however undergo surgical resection. As surgical resection is the treatment for both symptomatic ILS and ELS, there is no real difference in management. The main difference between the treatment of ILS versus ELS is that because extralobar sequestrations have a separate pleura, the sequestration can typically be removed while sparing normal lung tissue. Conversely, for intralobar sequestrations, segmental resection or lobectomy could be necessary, as there is no pleura separating the normal lung tissue from the sequestration.

## Conclusions

This case highlights the importance of considering pulmonary sequestration, especially intralobar sequestration in the differential diagnosis of recurrent pulmonary infections, especially in a patient who is otherwise healthy. CT imaging is the modality of choice for diagnosis, and surgical excision is curative. Clinicians should be aware of the possibility of future interventions if symptoms recur and should educate patients accordingly. Although rare, it is important to consider this congenital anomaly when evaluating otherwise healthy patients with recurrent pulmonary infections, chest pain, or hemoptysis.
